# Exploring a Structural Basis for Delayed Rod-Mediated Dark Adaptation in Age-Related Macular Degeneration Via Deep Learning

**DOI:** 10.1167/tvst.9.2.62

**Published:** 2020-12-15

**Authors:** Aaron Y. Lee, Cecilia S. Lee, Marian S. Blazes, Julia P. Owen, Yelena Bagdasarova, Yue Wu, Theodore Spaide, Ryan T. Yanagihara, Yuka Kihara, Mark E. Clark, MiYoung Kwon, Cynthia Owsley, Christine A. Curcio

**Affiliations:** 1 Department of Ophthalmology, School of Medicine, University of Washington, Seattle, WA, USA; 2 Department of Ophthalmology and Visual Sciences, School of Medicine, University of Alabama at Birmingham, Birmingham, AL, USA; 3 Department of Psychology, Northeastern University, Boston, MA, USA

**Keywords:** deep learning, age-related macular degeneration, biomarker, spectral domain optical coherence tomography, drusen, rod-mediated dark adaptation

## Abstract

**Purpose:**

Delayed rod-mediated dark adaptation (RMDA) is a functional biomarker for incipient age-related macular degeneration (AMD). We used anatomically restricted spectral domain optical coherence tomography (SD-OCT) imaging data to localize de novo imaging features associated with and to test hypotheses about delayed RMDA.

**Methods:**

Rod intercept time (RIT) was measured in participants with and without AMD at 5 degrees from the fovea, and macular SD-OCT images were obtained. A deep learning model was trained with anatomically restricted information using a single representative B-scan through the fovea of each eye. Mean-occlusion masking was utilized to isolate the relevant imaging features.

**Results:**

The model identified hyporeflective outer retinal bands on macular SD-OCT associated with delayed RMDA. The validation mean standard error (MSE) registered to the foveal B-scan localized the lowest error to 0.5 mm temporal to the fovea center, within an overall low-error region across the rod-free zone and adjoining parafovea. Mean absolute error (MAE) on the test set was 4.71 minutes (8.8% of the dynamic range).

**Conclusions:**

We report a novel framework for imaging biomarker discovery using deep learning and demonstrate its ability to identify and localize a previously undescribed biomarker in retinal imaging. The hyporeflective outer retinal bands in central macula on SD-OCT demonstrate a structural basis for dysfunctional rod vision that correlates to published histopathologic findings.

**Translational Relevance:**

This agnostic approach to anatomic biomarker discovery strengthens the rationale for RMDA as an outcome measure in early AMD clinical trials, and also expands the utility of deep learning beyond automated diagnosis to fundamental discovery.

## Introduction

Age-related macular degeneration (AMD) causes significant visual impairment and progressive loss of central vision in older adults.^1^ Although therapies are available for exudative AMD, the more common nonexudative form of AMD still lacks an effective treatment.^2^^,^^3^ Early biomarkers for nonexudative AMD are needed to advance clinical trials of potential therapies and to help identify patients at risk for progressing to advanced disease. Imaging biomarkers are favored for speed and objectivity; ideally, imaging biomarkers are also indices of visual function.

Delayed rod-mediated dark adaptation (RMDA) is a slower return to retinal sensitivity following a bright light flash stimulus and a known functional biomarker for incipient early AMD. Older patients with normal macular health who show delayed RMDA, measured as longer rod intercept times (RITs), have an increased risk of developing AMD.^4^^,^^5^ In addition, delayed RMDA has been associated with common polymorphisms of two major AMD-associated genes, complement factor H and the age-related maculopathy susceptibility 2 (ARMS2) gene.^6^ The idea that rod-mediated vision has merit for documenting the progression of macular disease is well supported. First, older adults in normal health report difficulty with visual tasks performed at low luminance levels.^7^ Second, comprehensive maps of photoreceptor density in human macula demonstrate not only a high density of foveal cones but also numerous rods.^8^ Expressed in units of the widely used Early Treatment Diabetic Retinopathy Study (ETDRS) grid, the central subfield contains almost exclusively cone photoreceptors, the inner ring (0.5–1.5 mm from the foveal center) has a 4:1 rod:cone ratio, and the outer ring (1.5–3 mm) has a 10:1 ratio.^9^ Eyes of aged donors exhibit loss of rods especially in the inner ring.^10^ Third, RMDA was proposed by Bird and Fitzke as a dynamic measure of retinoid resupply to rods across the choriocapillaris-Bruch's membrane-retinal pigment epithelium (RPE) interface,^11^ where AMD pathology is prominent, and given a strong neurophysiologic underpinning by Lamb and Pugh.^12^ Fourth, documented cellular and molecular age changes in the retinoid resupply route include loss of macular choriocapillaris and lipidization of Bruch's membrane due to retention of lipoproteins of intraocular origin, while RPE cell numbers are maintained, suggesting a vascular-originating degeneration.^13^^–^^15^ In contrast, cone-mediated visual acuity in bright light can remain preserved well into the disease course, attributed to additional sustenance by foveal Müller glia.

Early signs of AMD, as revealed by color fundus photography, can also be detected on spectral domain optical coherence tomography (SD-OCT). Normal outer retinal structure shows bands of varying reflectivity on SD-OCT due to horizontally aligned, vertically compartmentalized photoreceptors and supporting RPE and glia.^16^^,^^17^ In early and intermediate AMD, hyper-reflective foci (clumps) in the retina are associated with both progression risk and delayed RMDA.^18^^–^^20^ Recent studies clearly linked aberrant imaging findings in imaging to histopathologic changes.^20^^,^^21^ However, biomarkers for even earlier stages of disease may require a different strategy. The intricacy and small size of outer retinal cells challenge histologic quantification of anatomy due to disorganization during postmortem processing, including problems discerning the lengths of photoreceptor inner and outer segments. Thus, SD-OCT images of the human retina in vivo have the potential to answer such questions. In addition, human eyes are advantageous for studying AMD over laboratory animals lacking maculae. Even monkey eyes that do have maculae and develop drusen are less overall rod-dominant than humans and do not progress to AMD end-stages.^22^^,^^23^

Advances in artificial intelligence may provide novel methods for identifying anatomic features on SD-OCT that correlate with a known measure of retinal dysfunction. Deep learning algorithms in particular offer a unique approach to this challenge. Unlike automated diagnostic machine learning models,^24^^–^^28^ which are trained using hand-labeled data to detect known findings, supervised deep learning models can also be trained to identify image characteristics that correspond to a known measurement in a previously unrecognized way.^29^^,^^30^ Using functional, objective training targets with visualization techniques, a deep learning model could potentially identify novel imaging features and specific anatomic details on SD-OCT that correlate with a known functional biomarker, such as delayed RMDA. In this study, we sought to train deep learning models to predict the rate of RMDA using RIT and anatomically restricted SD-OCT imaging data as well as localize de novo imaging features associated with RMDA.

## Methods

This study was approved by the Institutional Review Board of the University of Alabama at Birmingham, followed the tenets of the Declaration of Helsinki and was conducted in compliance with the Health Insurance Portability and Accountability Act. Informed consent was obtained from all subjects. The collection of data for the Alabama Study on Early Age-Related Macular Degeneration has been described previously.^4^^,^^31^

SD-OCT volumes were obtained with Spectralis HRA + SD-OCT (Heidelberg Engineering, Heidelberg, Germany). We acquired SD-OCT volumes of all maculae (Spectralis HRA + SD-OCT; Heidelberg Engineering). B-scans (*n* = 73) were horizontally oriented and centered over the fovea in a 20 degree × 15 degree (5.7 × 4.2 mm) area. Automatic Real-Time averaging was 8 to 18, and quality was 20 to 47 dB. All SD-OCT images shown in this paper are unadjusted from the original manufacturer's intensities.

Dark adaptation was measured at 1-2 visits, using the AdaptDx (MacuLogix, Harrisburg, PA) adaptometer in a 20 minute test protocol, which has been described^4^^,^^32^ and validated^33^ in previous studies. Briefly, patients’ eyes were dilated to ≥ 6 mm and the non-test eye was occluded. The test eye was aligned to a red fixation light using an infrared camera system, and a focal photoflash (0.25 ms duration, 58,000 scotopic cd/m^2^ second intensity; equivalent approximately 85% bleach) centered at 5 degrees on the superior vertical meridian was applied for bleaching. Targets were then presented to this area every 2 to 3 seconds (beginning at 5.00 cd/m^2^) and decreasing in intensity by steps (0.3 log units), and patients responded by pressing a button when they saw a stimulus light until they could no longer detect them. The stimulus light intensity then increased in small (0.1 log unit) increments, and the intensity at which the patient was able to detect the light once again was recorded. The RIT was defined as the duration after the photo bleach required for sensitivity to recover to a stimulus light sensitivity of 5 × 10^−4^ cd/m2, which is located within the second component of rod recovery.^12^ No subjects were excluded due to fixation loss or poor reliability. The RIT and the OCT images were captured on the same day or within 1 week.

**Figure 1.**
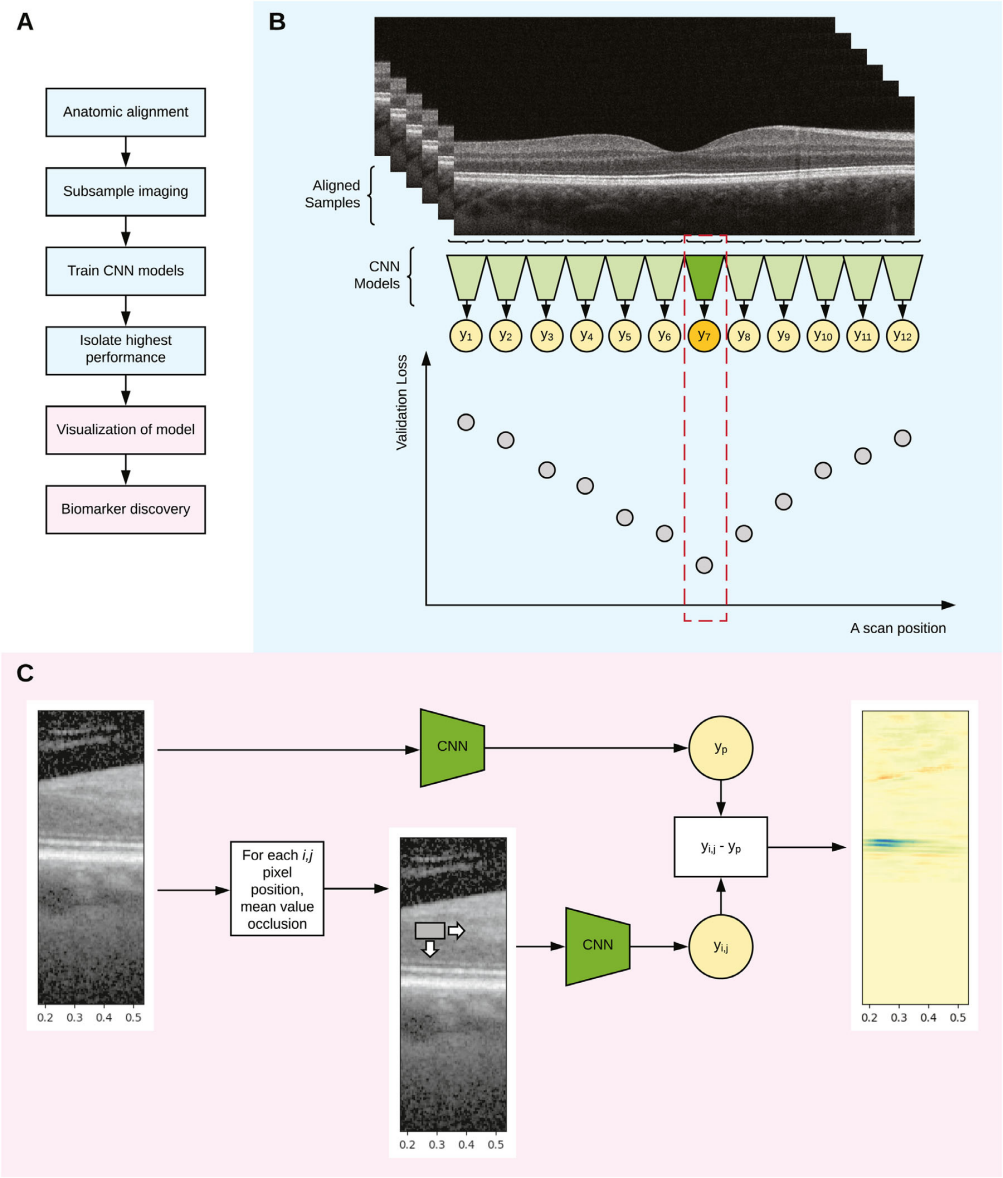
Concept diagram of framework for biomarker discovery using deep learning. The overall framework is shown in (**A**). After aligning spectral domain optical coherence tomography (SD-OCT) images, separate datasets are created and different convolutional neural networks (CNN) deep learning models are trained (**B**). The frozen models with the best performance, lowest validation loss, are systematically perturbed with mean occlusion in the test set and perturbations increasing and decreasing the predictions are shown in *green* and *red*, respectively (**C**).

The dataset was partitioned into three mutually exclusive sets at the patient level for training (60%), validation (20%), and test (20%). A conceptual framework for processing and localizing biomarkers was developed (Fig. 1). This framework consisted of two parts. First, the SD-OCT volumes were anatomically registered and deep learning models were separately trained on narrow bands of the B-scan that passed through the foveal center of each eye, where each band was centered at an anatomic location (see Fig. 1B). Second, the vertical B-scan window that corresponded to the anatomic location with the highest performance was then extracted (see Fig. 1B) and systematically perturbed to find the areas leading to higher or lower predicted RIT using the test set (see Fig. 1C). Only B-scan vertical windows were used to train the models. The number of B scans per volume did not differ between subjects.

In part one, one B-scan that was found to pass through the foveal center was extracted from each volume. At each anatomic location in this foveal B-scan, a 64 × 256 pixel window was created such that the anatomic location was in the middle of the window and the vertical placement of the window was placed on the horizontal maximum intensity projection. A deep learning model was trained for each anatomic location using the same neural architecture (Supplementary Figure S1). The input to the model was the raw pixel intensities divided by 255, without other normalizations or transformations from the 64 × 256 pixel window, and the output for the model was a single node with linear activation to predict the RIT divided by 40 minutes from the RMDA testing to scale the output of the models between 0 and 1. Mean squared error was used as the loss function. Weights for the convolutional layers were initialized randomly using the Xavier normal distribution.^34^ Nesterov Adam^35^ was used as an optimizer with an initial learning rate of 2 × 10^−4^. Batch size was set to 26 and the number of epochs was set to 600. For each anatomic position, nine repetitions of the training session were performed to account for different random initializations because the main outcome of the study was to evaluate the model capacity to learn to predict the RIT. Each training session used a fixed set of hyperparameters. The validation losses were collected and the weights of models from the lowest validation loss of each training session were saved for further analysis (see Fig. 1B).

For part two, test-set vertical B-scan images from the anatomic location with the lowest loss were used for visualization of relevant features. At each pixel position in the image for the whole OCT B-scan, a 16 × 6 pixel window was occluded using the mean of the pixel intensity of the window and inference was performed. Occlusion using permuted pixel intensities, zero intensity, different window sizes led to similar results. The difference in predicted RIT between the model output of the altered image and the unaltered image was measured for an occlusion window centered at each pixel position. A color map with the difference in predicted perturbed RIT from the unperturbed RIT was then plotted to visualize both lengthening and shortening perturbations on the OCT B-scan (see Fig. 1C). These differences were then evaluated qualitatively using a random sampling method in the test-set.

All analyses were performed using Python (version 2.7.12) and R (version 3.3.2). Deep learning models were developed using Keras (version 2.2.0), Tensorflow (version 1.7.0), accelerated using NVIDIA CUDA (version 9.0.333), and trained on a server with dual Xeon 3.4 GHz processors, 256 GB of random access memory, and 8 x NVIDIA P100 GPUs.

## Results

Seven hundred fifteen patients were imaged using SD-OCT and tested for RMDA. RIT was measured in 737 eyes in 1-2 visits, resulting in 1218 OCT volumes of individual eyes paired with RIT measurements. The demographics of this population are shown in the Table 1.

**Table.** Patient Characteristics

	Training	Validation	Test	Total
Patients, *n*	424	148	143	715
Gender, *n*				
Male	147	57	56	260
Female	277	91	87	455
Race				
White	405	145	130	680
African American	15	3	15	28
Asian	2	0	1	3
Other	2	0	2	4
Eyes, *n*	436	154	147	737
SD-OCT volumes, *n*	711	254	253	1218
Age, mean (SD)	70.9 (6.4)	71.0 (6.3)	71.5 (6.2)	71.0 (6.3)
AREDS (Grade) Category, *n*				
Normal (1)	482	170	173	825
Early (2–4)	167	70	57	294
Intermediate (5–8)	47	13	19	79
Advanced (9–11)	15	1	4	20
Rod intercept time minutes, mean (SD)	13.2 (9.0)	12.9 (8.4)	13.3 (10.3)	13.1 (9.2)

Demographic information and age-related macular degeneration disease severity of study participants.

AREDS, Age-Related Eye Disease Study; SD, standard deviation; SD-OCT, spectral domain optical coherence tomography.

**Figure 2.**
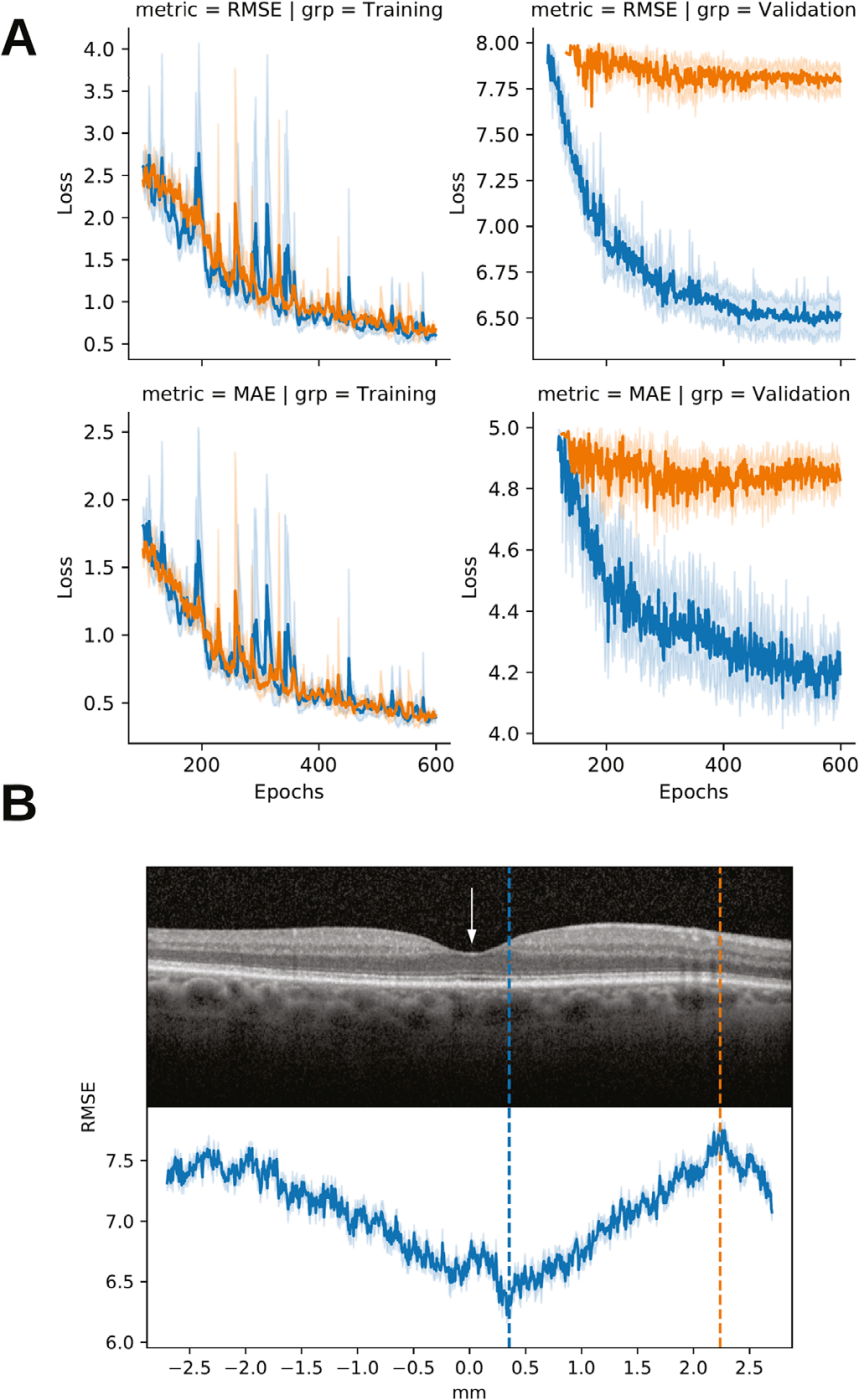
Performance of deep learning models by anatomic location. Training curves for two different anatomic locations (*blue and orange curves*) (**A**) by root mean standard error (RMSE) and mean absolute error (MAE); shaded region shows 95% confidence intervals by repeated training sessions. The anatomic positions are indicated by the two dotted lines of corresponding color in panel (**B**). Lowest error on foveal B-scan by millimeters eccentricity and RMSE loss with lower being higher performance **B**. The fovea is labeled with the *white arrow*.

Convolutional neural networks were independently trained on each of the narrow SD-OCT bands at various anatomic locations. At each anatomic location, the model was trained nine times with newly randomized initialized weights, and the weights corresponding to the lowest root mean squared error (RMSE) in the predicted RIT were chosen out of each training session. The RMSE and mean absolute error (MAE) of the nine models at each anatomic position were averaged (Fig. 2A) and collected as a function of eccentricity from the fovea in mm (see Fig. 2B). The trained models achieved an overall MAE across all the bands in the test set of 4.71 minutes for predicting RIT (8.8% of the dynamic range and lower than normal upper bound of 12.3 minutes).^4^ In the test set, the correlation between the predicted RIT and the true RIT showed moderately high correlation (Pearson's correlation of 0.69).

Figure 2B plots RSME across an entire foveal B-scan. This curve decreases smoothly from highest values at approximately 2 mm eccentricity to lowest values (i.e. most accurate RIT predictions), at 0.34 mm within a central area 1 mm in diameter (0.5 mm radius, or eccentricity from the foveal center). This region corresponds to the central subfield of the ETDRS grid, commonly used in clinical and epidemiologic studies,^36^ and includes the all-cone fovea (350 µm diameter) with a rim of low rod density. In the macular retina immediately surrounding the fovea, cone density steadily declines and rod density steadily increases.^8^

Using images from the test set centered on 0.34 mm (1.2 degrees) nasal eccentricity, the relative impact of systematic mean-occlusion based perturbations were assessed on the trained models. For each pixel position, an occlusion mask was placed using the mean value of the occlusion window, and inference was performed, where the predicted RIT was the nine-model ensemble average. The deep learning model dependence on specific SD-OCT features was identified by systematically perturbing the input images and testing the model error. The resulting differences in the RIT predictions were compared against the baseline RIT prediction without perturbations, identifying specific SD-OCT signatures that the models were most dependent upon for predicting RIT. Because RIT is a continuous value, both the direction and magnitude of the perturbed inferences were measured. The specific SD-OCT signatures of different areas that caused a longer, more pathologic RIT were identified.

**Figure 3.**
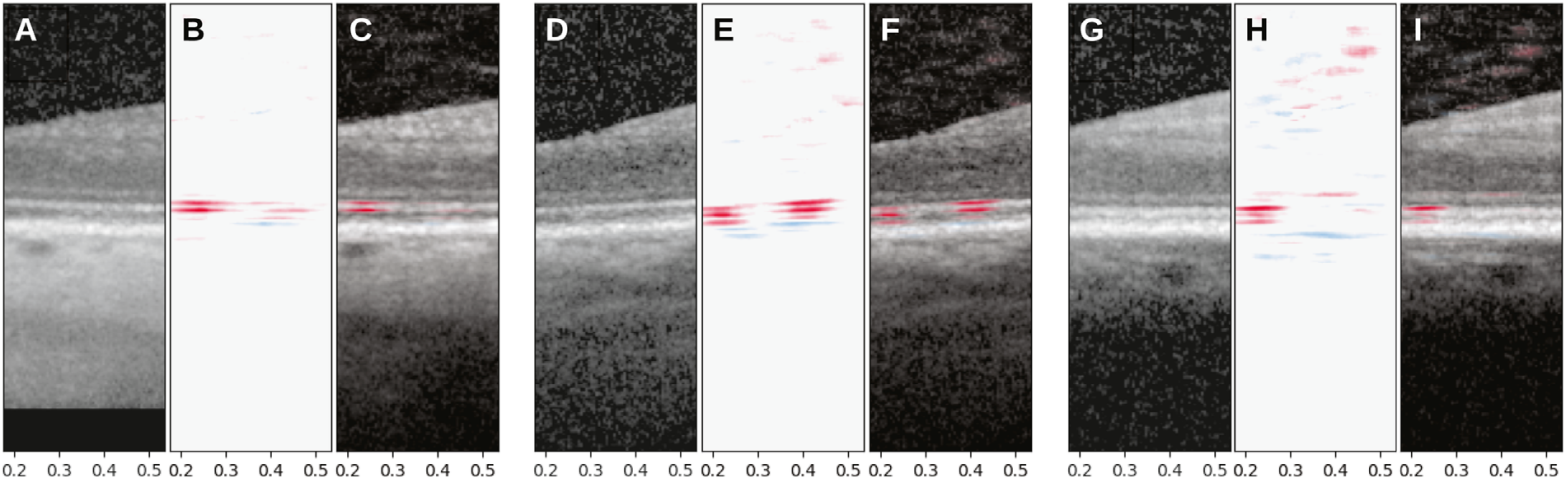
Visualization of deep learning features from the test set. The original spectral domain optical coherence tomography (SD-OCT) scan in mm used by the deep learning model to predict rod intercept time (RIT) are shown in (**A, D, G**). Panels (**B, E, H**) show the magnitude of the difference between the perturbed and baseline predictions caused by occlusion of each possible pixel position, with *red* showing elongation and *blue* showing shortening of the RIT. The corresponding overlays are shown in (**C, F, I**) in relation to the ellipsoid zone (EZ).

**Figure 4.**
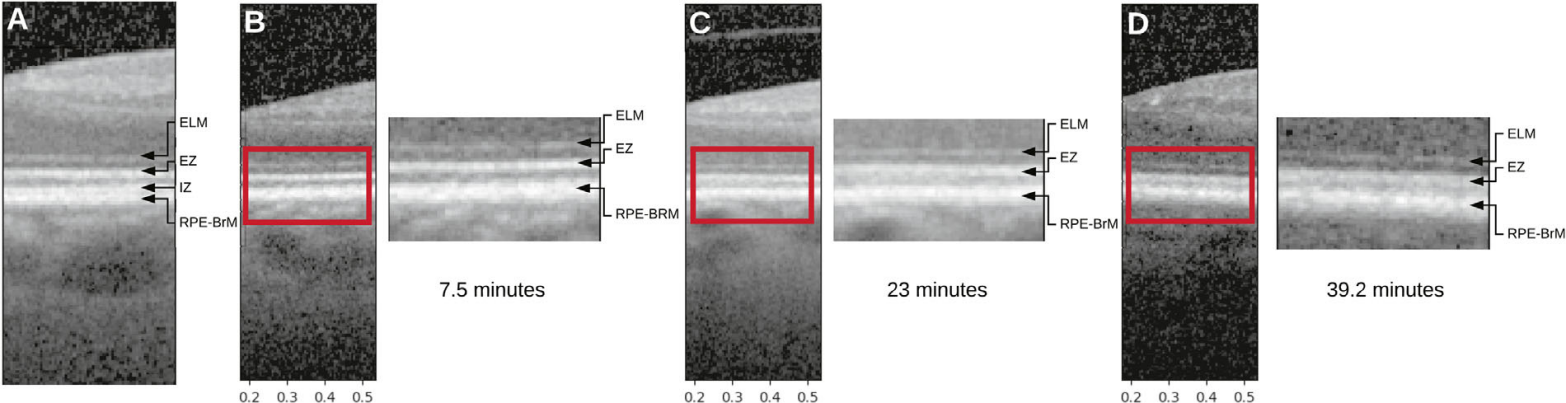
Correlation of hyporeflective bands with rod intercept time (RIT). Panel (**A**) shows a reference image of the external limiting membrane (ELM), the ellipsoid zone (EZ), the interdigitation zone (IZ), and the retinal pigment epithelium-Bruch's membrane (RPE-BrM) on spectral domain optical coherence tomography (SD-OCT). Three examples of low RIT (**B**), medium RIT (**C**), and high RIT (**D**) sampled randomly from the test set are shown with high resolution insets (*red boxes*) and the RIT in minutes. The IZ, which is apparent in the reference figure (also from this population), is not apparent in any of the randomly sampled figures. Further the gap between the RPE-BrM and the EZ is more hyper-reflective in **C**, **D** than in **B**. Blurring of hyporeflective bands superficial and deep to the EZ correlates with RIT.

Visual observation of the test set results showed that the model was reliant on two hyporeflective regions that bounded the ellipsoid zone (EZ) of the inner segment (Fig. 3). The first region of interest represents the myoid portion of the photoreceptor inner segments (above the EZ) and the second represents a relatively hyporeflective area below the EZ and above the interdigitation zone (IZ), between the outer segment tips and the RPE apical processes (below the EZ).^37^ The areas associated with an increase of RIT were in the myoid zone, whereas areas associated with a decrease of RIT were localized to apical and basal aspects of the RPE cell body, possibly including choriocapillaris basally. Examination of high-resolution extractions from these areas in the test set revealed that subtle reflectivity changes in these areas were correlated with the RIT (Fig. 4).

## Discussion

Advancements in artificial intelligence with the advent of deep learning have revolutionized biomedical image analysis. Although many applications of deep learning models have been shown to reach expert level consensus for automated diagnosis and feature segmentation, current applications mainly recapitulate human understanding of diseases. In our study, we demonstrate a novel framework for localization and identification of biomarkers using deep learning by first restricting information to anatomically registered locations and subsequently applying visualization techniques. By training a deep learning model with 1218 SD-OCT volumes paired with RMDA measurements, we isolated the relevant imaging features corresponding to the RMDA in a label-agnostic way. Thus, we discovered hyporeflective outer retinal bands, in a specific topography, as a novel structural basis for a functional biomarker of incipient AMD. Our use of deep learning in this study is additionally novel relative to recent publications in ophthalmology by focusing on mechanistic questions rather than automated diagnosis.^25^^,^^38^^–^^42^ Interestingly, our findings point to specific laminar and topographic correlations within the retina for delayed RMDA, over and above person-level associations, such as genetic predisposition^6^ and plasma metabolites.^43^^–^^46^

Human retina at the interface of aging and early AMD is especially suited for the demonstrated framework of novel biomarker discovery. The eye is the brain's camera, and in it the photoreceptors and supporting cells (glia, vascular endothelium, and RPE) are deployed with high geometric precision similar to a charge-coupled device chip. Comprehensive histologic mapping studies show that humans have a cone-only fovea with the point of highest cone photoreceptor density in the foveal center.^8^ Rods are absent from this center. In young adults, rods outnumber cones 4:1 at 0.5-1.5 mm eccentricity^9^ and crest in an elliptical ring at 3 to 5 mm, encircling the optic nerve head. In eyes of older adults like those included in our data, cones are stable in number and rods decline 30% in the 0.5 to 1.5 mm ring, exactly including the location of our biomarker.

What are the anatomic correlates of the OCT signatures identified as associated with delayed RMDA? Each reflective band in the outer retina represents the precise horizontal alignment of vertically compartmentalized cells (i.e. photoreceptors, RPE, and Müller glia).^47^ One prominent landmark is the hyperreflective EZ, which in commercial SD-OCT represents the mitochondria-rich ellipsoid of photoreceptor inner segments. In Figure 3, the EZ is flanked above and below with hyporeflective bands that are predictive of RIT. The upper of these hyporeflective bands identified in this study represents the myoid portion of photoreceptor inner segments, and is identified as the “myoid zone of the photoreceptors” in the SD-OCT clinical lexicon.^37^ This part of the cell contains Golgi apparatus and ribosomes and is known to shorten in AMD.^48^ The lower of the two hyporeflective bands (visible in Fig. 3 but not in Fig. 4) is unnamed in the clinical lexicon, but is located below the EZ and above the IZ, between the outer segment tips and the RPE apical processes. The IZ is a hyper-reflective band that involves photoreceptor outer segments and the delicate apical processes of the RPE, which contain melanosomes.^49^ The difference between Figures 3 and 4 suggests that in many eyes with longer RIT, the region between the EZ and RPE-Bruch's membrane bands (containing both this unnamed hyporeflective region of interest and the IZ) becomes variably reflective, shorter, or both, thus blunting distinctions between the EZ and the apical RPE on OCT imaging. Whether this is due to altered apical processes or outer segments or both cannot be determined with the resolution of these SD-OCT images. Other OCT technologies with greater axial resolution^50^ may be useful in addressing this question.

The regional specificity of the discovered biomarker near the fovea deserves comment, in two ways. First, the SD-OCT B-scan used for training did not pass through the retinal location used for RMDA testing (5 degrees or 1.44 mm superior to the fovea, on the vertical meridian). Second, the most accurate anatomic correlates for rod-mediated vision included the fovea, an area containing only cones, and extended to the adjoining rod-dominant parafovea. These seeming contradictions occur, because rods at the RMDA testing location are impacted by pathologic changes in the central macular region identified by the model.

RMDA is a readout of age-related changes and pathology in the underlying choriocapillaris endothelium and Bruch's membrane, representing the retinoid resupply route from the circulation. A sequence of age-related changes in these support tissues leading to soft drusen and advanced AMD pathology have been elucidated by ultrastructural studies, histochemistry, lipid profiling, gene expression, cell biology, and in vivo clinical imaging. This sequence is most prominent under the fovea, with a spread within the central 3 mm diameter of the macula that includes precisely the area identified by the deep learning model. In fact, compared with other retinal locations, the presence and growth of drusen concentrated under the fovea have the greatest effect size in predicting 10-year risk of neovascularization or atrophy (relative risk at 10 years = 26.5 for baseline drusen in central 1 mm; 8.6 for drusen at 0.5–1.5 mm eccentricity).^51^ An Oil Spill model of drusen formation has been recently proposed^52^^,^^53^ as a late-life sequela of plasma high density lipoproteins (HDLs) delivering xanthophyll carotenoid pigments (lutein and zeaxanthin) to foveal cells, in particular the Müller glia that extend processes laterally within the inner and outer plexiform layers.^54^ Cones themselves are sustained by these Müller glia, which are in turn supported by retinal capillaries at the edge of the avascular zone. The rods are relatively vulnerable, because they are more dependent on the choriocapillaris Bruch's RPE than are the cones. This hypothesis integrating drusen biology with retinal neuroscience^52^^,^^55^ to explain both rod vulnerability and cone resilience^9^ incorporates multiple evidence lines from human biology, including sequence variants in AMD-associated HDL genes.^46^ Parts of this hypothesis remain to be confirmed, and it does not exclude mechanisms with pan-retinal or systemic underpinnings (e.g. inflammation). It does emphasize a local-ness of AMD dysfunction that is best explained by heretofore unrecognized aspects of outer retinal cell physiology.

Strengths of the current study include the use of a functional training target with deep learning models as opposed to human expert derived disease classifications, and a de novo, agnostic approach to image analysis. The use of RIT from RMDA testing allowed discovery of previously undescribed biomarkers rather than simply recapitulating human understanding of disease. The study patients were drawn from a carefully selected cohort spanning aging and early AMD disease severities allowing for investigations into the pathophysiology of AMD. If there were no association between eccentricity and the capacity of the deep learning models to predict RIT, a flat line would have been observed in Figure 2B. Instead, a gradual increase in predictability was found centrally, and the choice of the foveal B-scan allowed for unbiased hypothesis testing outside of the RMDA stimulus region. It is important to note that our deep learning framework shows for the first time in vivo the retinal area of interest that precisely matches the topography of age-related rod loss that was discovered histologically over several decades ago.^8^ In addition, it also validates the idea that rods near the fovea, which are not widely appreciated, are sensitive indicators of their support system, critical in diagnosing and understanding the pathophysiology of early AMD.^9^ Testing existing theories of disease are crucial for advancing our knowledge of AMD and allowing future therapeutic options; the agnostic nature of deep learning is particularly suitable for this task.

Limitations of this study include the possibility that more than one area is important in a single B-scan through the fovea for predicting RIT. In the first step of the analysis, the areas were restricted to narrow windows of the foveal B-scan to allow deep learning models to have access to high-resolution information. Although one way to circumvent this limitation is to train models with the full B-scan image, the current limitations in computer hardware prevent using the full image at native resolution for training. Aggressive downsampling may limit biomarker identification. In addition, the RMDA stimulus area was set to an area outside of the foveal B-scan. As with many biomarkers, the discovered features may correlate with the RIT instead of being directly indicative of disease initiation in the choriocapillaris-Bruch's membrane complex. The deep learning model is limited by the resolution of the input images and therefore may miss subtle changes to outer segments or RPE apical processes in this early disease population. We chose mean occlusion as the visualization method utilized in this study, because many other methods developed are designed for classification problems.^56^^,^^57^ Whereas occlusion methods are sensitive to the window size and occlusion value, we performed sensitivity analyses that showed that the biomarker was robust to these choices. Similarly, the discovered features do not correlate with the location of subretinal drusenoid deposits, an extracellular deposit most abundant at eccentricities only partly captured by the SD-OCT volumes studied here and associated with markedly increased vision loss at more advanced disease stages than present in this study population.^33^^,^^58^ The blue lines in Figure 3 are weaker and less consistent than the red bands and they may be more prominent in an image dataset derived from a different study design. The scanning parameters used for the data are another potential limitation, as models are sensitive to the distribution of the training input signal. Because the goal of this study was to identify a biomarker internally within our dataset, the parameters should not have affected our results. Future studies may need to include a wider range of OCT scanning parameters. Finally, although our model did not show a performance that could be clinically useful, the model predictions did show moderately high correlation in the test set and enabled uncovering new biomarkers.

Future work includes replication and longitudinal validation of this biomarker in external datasets and the application of this framework to other human diseases. In conclusion, we have demonstrated a new framework for discovery of biomarkers in human diseases using deep learning and applied this framework to AMD using the RIT from RMDA testing, and discovered a novel biomarker de novo. This biomarker fits with current concepts of AMD pathophysiology by highlighting both the topography and a structural basis for a functional biomarker (RIT). Establishment of biomarkers for the most common form of AMD where currently limited therapy is available will lead to more sensitive imaging-based clinical end points, an acceleration of clinical trials, and new therapeutic interventions. By confirming RMDA is closely linked to processes in the choriocapillaris-Bruch's membrane-RPE complex that lead to advanced disease, its use as an outcome measure is supported.

## Supplementary Material

Supplement 1
