# Supplementary material for: Exploring a Structural Basis for Delayed Rod-Mediated Dark Adaptation in Age-Related Macular Degeneration Via Deep Learning
Source: Transl Vis Sci Technol. 2020 Dec 15;9(2):62. doi: 10.1167/tvst.9.2.62 (PMC7745629; doi:10.1167/tvst.9.2.62)
Supplement: Supplement 1 [file tvst-9-2-62_s001.pdf]

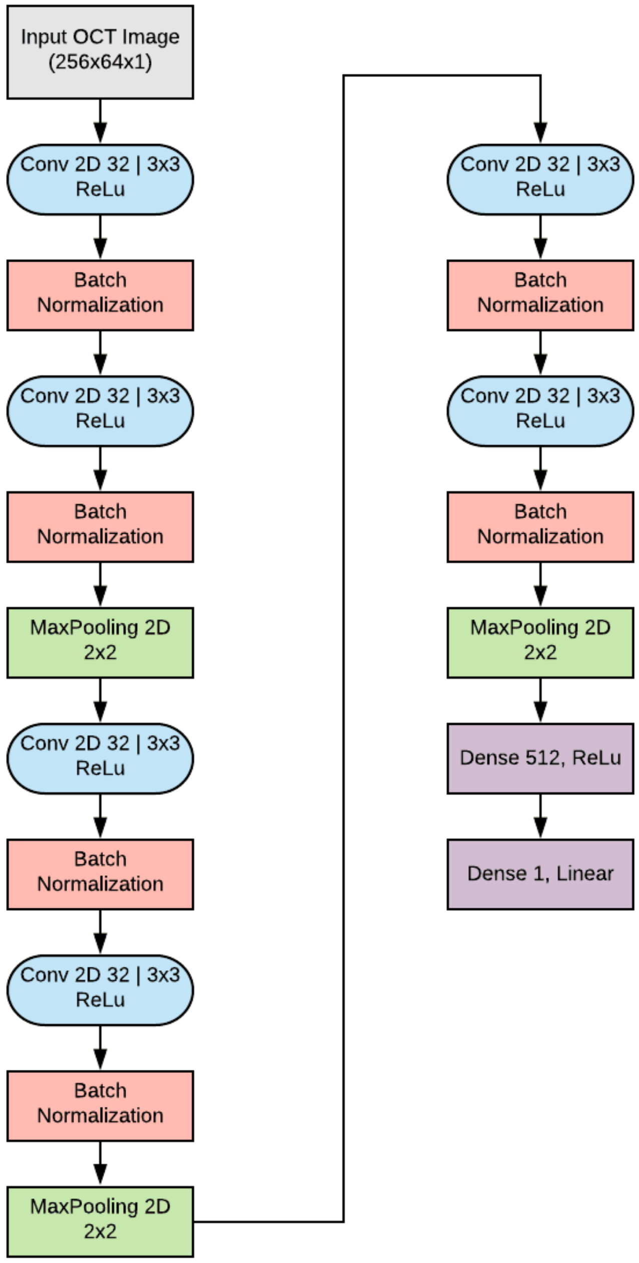

Supplemental Figure 1. Model architecture used for training. Strides were set to 1 for all convolutional filters and maxpooling steps. The number of convolutional filters at each step is shown along with the filter sizes. The output of the model is the predicted rod-mediated dark adaptation rod intercept time. The loss used is mean squared error with a batch size of 26 and Nesterov-Adam optimizer with a learning rate of 2 x 10-4.
